# Reproducibility and repeatability of a semi-automated pipeline to quantify trapeziometacarpal joint angles using dynamic computed tomography

**DOI:** 10.1186/s12880-022-00922-2

**Published:** 2022-11-08

**Authors:** Michael T. Kuczynski, Kendra Wang, Justin J. Tse, Tomasz Bugajski, Sarah L. Manske

**Affiliations:** 1 Biomedical Engineering Graduate Program, Schulich School of Engineering, University of Calgary, Calgary, Canada; 2 McCaig Institute for Bone and Joint Health, University of Calgary, 3280 Hospital Drive, N.W., Calgary, AB T2N 4Z6 Canada; 3 Department of Radiology, Cumming School of Medicine, University of Calgary, Calgary, Canada; 4 Biomedical Engineering Undergraduate Program, University of Waterloo, Waterloo, Canada

**Keywords:** Joint angle, Cadaver, Dynamic computed tomography, Trapeziometacarpal joint

## Abstract

**Background:**

The trapeziometacarpal (TMC) joint is a mechanically complex joint and is commonly affected by musculoskeletal diseases such as osteoarthritis. Quantifying in vivo TMC joint biomechanics, such as joint angles, with traditional reflective marker-based methods can be difficult due to the joint’s location in the hand. Dynamic computed tomography (CT) can facilitate the quantification of TMC joint motion by continuously capturing three-dimensional volumes over time. However, post-processing of dynamic CT datasets can be time intensive and automated methods are needed to reduce processing times to allow for application to larger clinical studies. The purpose of this work is to introduce a fast, semi-automated pipeline to quantify joint angles from dynamic CT scans of the TMC joint and evaluate the associated error in joint angle and translation computation by means of a reproducibility and repeatability study.

**Methods:**

Ten cadaveric hands were scanned with dynamic CT using a passive motion device to move thumbs in a radial abduction–adduction motion. Static CT scans and high-resolution peripheral quantitative CT scans were also acquired to generate high-resolution bone meshes. Abduction–adduction, flexion–extension, and axial rotation angles were computed using a joint coordinate system. Reproducibility and repeatability were assessed using intraclass correlation coefficients, Bland–Altman analysis, and root mean square errors. Target registration errors were computed to evaluate errors associated with image registration.

**Results:**

We found good repeatability for flexion–extension, abduction–adduction, and axial rotation angles. Reproducibility was moderate for all three angles. Joint translations exhibited greater repeatability than reproducibility. Specimens with greater joint degeneration had lower repeatability and reproducibility. We found that the difference in resulting joint angles and translations were likely due to differences in segment coordinate system definition between multiple raters, rather than due to registration errors.

**Conclusions:**

The proposed semi-automatic processing pipeline was fast, repeatable, and moderately reproducible when quantifying TMC joint angles and translations. This work provides a range of errors for TMC joint angles from dynamic CT scans using manually selected anatomical landmarks.

## Background

The trapeziometacarpal (TMC) joint at the base of the thumb provides the unique prehensile ability of the hand, allowing for grasping and pinching. Defined as a biconcave-convex or saddle shaped joint [1] the TMC joint is supported by 16 ligaments [2] and has been shown to experience contact forces of up to 120 kg during strong grasps [3]. The biomechanical complexity of the TMC joint increases the joint’s predisposition to the development of joint diseases such as osteoarthritis (OA), wherein ligament laxity [4] and repetitive motions [5–7] have been shown to increase the risk of development of OA. As such, better understanding the biomechanical behaviour of the TMC joint may provide new information in the study of diseases of the hand. However, the quantification of joint biomechanics in the TMC joint is often difficult in vivo due to the small size of bones and inaccessibility for skin-based marker placement (*e.g.*, carpal bones). To quantify joint motion in three dimensions (3D) in motion capture systems, reflective skin-based markers are placed on each bone segment in three spatial planes. However, non-invasively placing reflective markers on the TMC joint is often not possible, particularly for the trapezium located in the wrist. The use of non-invasive medical imaging technologies, such as computed tomography (CT), can provide 3D representations of joints. While previous imaging studies have shown changes in joint space and contact area [8, 9] in OA of the TMC joint, these studies have been limited to static imaging, which may not represent true joint biomechanics, particularly in diseased joints. Capturing true dynamic TMC joint motion may improve our understanding of TMC joint biomechanics and how altered joint biomechanics may relate to the development of diseases such as OA. Dynamic CT is a technique that has been commonly used in cardiac and respiratory imaging and is now emerging as a potential technique for imaging joint biomechanics in vivo. Dynamic CT provides 3D images over time, allowing for more accurate estimates of hand joint kinematics than is possible with motion capture systems. Several recent studies have utilized dynamic CT for various musculoskeletal applications and have demonstrated the feasibility of dynamic CT imaging for measuring joint motion (*i.e.*, rotations and translations), displacements, and even estimates of joint contact [10–14]. However, post-processing methods for dynamic CT datasets published to date are time intensive, reducing the practicality of applying this technique to larger clinical studies. In one study, 120 h were required to process a single TMC joint for a single movement [11], highlighting the need for fast, automated tools to process data from dynamic CT. Dynamic CT protocols typically suffer from low spatial resolution when compared to static CT scans, requiring additional post-processing to overcome these limitations. These additional post-processing steps often introduce new sources of error from image processing techniques, such as image registration between dynamic CT frames or registration between static, high-resolution CT images to dynamic CT frames.

When describing joint motion, segment coordinate systems (SCS) must first be defined. Using 3D bone meshes obtained from CT imaging, SCSs can be defined using points placed on anatomical landmarks (AL) to define each axis of the SCS, similar to the process used in motion capture systems. Previous motion capture studies using skin-based reflective markers have shown that the subjective task of manually placing markers on ALs can cause large variations in the resulting joint angle measurements [15]. These errors are often due to skin motion artifacts and inconsistencies between raters. While placing ALs directly on the bony anatomy in dynamic CT datasets removes the effect of skin motion artifacts, inconsistencies between raters may still produce errors in resulting joint biomechanics. Thus, the error associated with manually placed ALs in dynamic CT datasets warrants further investigation.

The purpose of this study was to introduce a fast, semi-automated pipeline to measure TMC joint angles from dynamic CT scans, and to evaluate the repeatability and reproducibility of manual AL placement on resulting joint angles. As a secondary analysis, we evaluated the effect of manual AL placement on the repeatability and reproducibility of joint translations. For this study, we define the repeatability as the precision of resulting joint angles and translations after repeated AL placement by a single rater. Reproducibility was defined as the precision of resulting joint angles and translations after AL placement by three separate raters. It was hypothesized that manually placed ALs will provide excellent repeatability and moderate reproducibility in resulting joint angles and translations.

## Materials and methods

### Specimen preparation

**Fig. 1:**
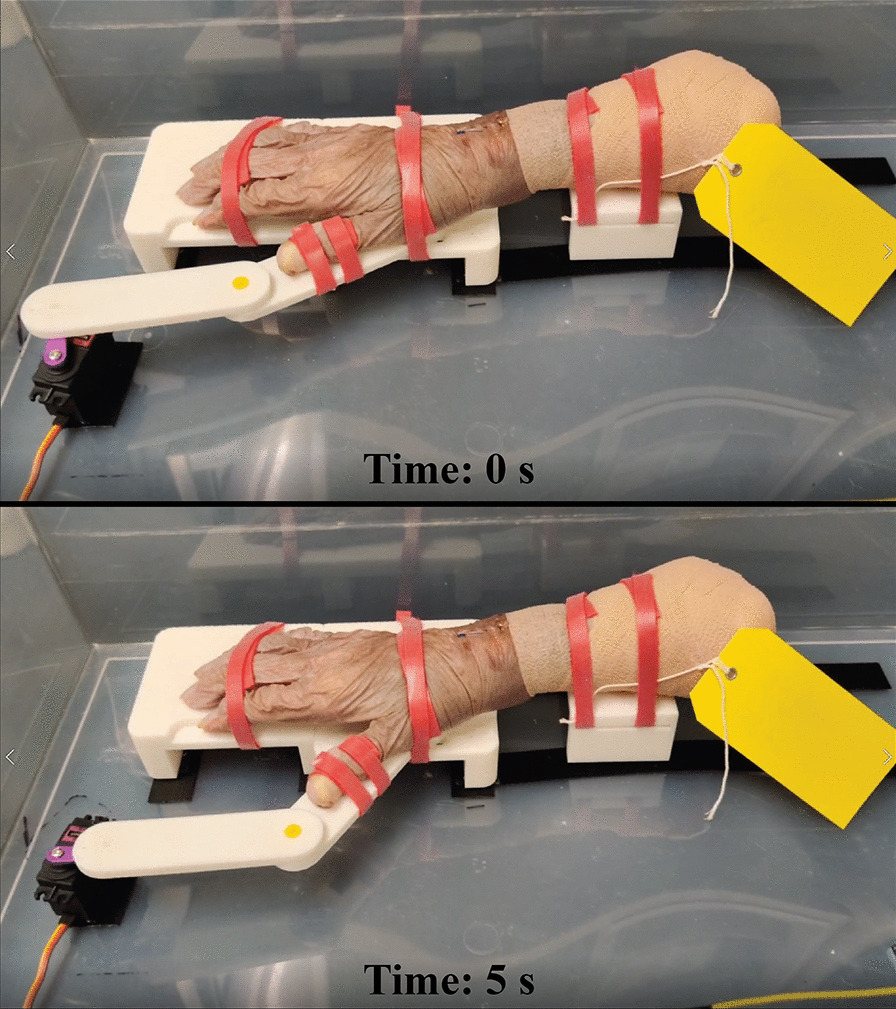
The passive motion device designed to move thumbs of each specimen in a radial abduction–adduction movement. An Arduino Uno microcontroller powers a servo motor to move the thumb through the range of motion. The main device platform was 3D printed

Ten cadaveric hands were acquired from the Advanced Technical Skills Simulation Laboratory at the University of Calgary (right hands, 4 female, 6 male, mean age: 81.6 ± 13.9 years). Each specimen contained the entire hand, wrist, and a portion of the forearm (Fig. 1). The medical history of each specimen was not obtained for this study. All methods were performed in accordance with guidelines set by the Conjoint Health Research Ethics Board at the University of Calgary (REB20-0039).

### Experimental setup

Prior to scanning, each specimen was securely strapped onto a custom 3D-printed passive motion device designed to move the thumb in a radial abduction–adduction motion (*i.e.*, functional flexion–extension) (Fig. 1). An Arduino Uno microcontroller powered a servo motor using software developed using C++ (Arduino IDE, v1.8.13). A resting position was defined as laying all fingers flat, pointing forward, and pressed together to minimize space between fingers. From several pilot scans, a time of 5 s for the thumb to move from a resting position to a fully abducted position and back to resting position, was determined to qualitatively minimize motion artifact. Range of motion was not standardized between specimens as we were only interested in errors associated between and within raters, not errors due to the passive motion device.

### Imaging protocol

Each specimen underwent dynamic CT scanning (GE Revolution HD GSI scanner, GE Healthcare, Chicago, USA) at the Centre for Mobility and Joint Health, University of Calgary. Dynamic CT scans were performed using 120 kVp, 100 mA, 0.4 s/gantry revolution, 40 mm longitudinal coverage, and 0.625 × 0.625 × 2.5 mm voxel size. Each specimen was scanned for 15 s, resulting in approximately three radial abduction–adduction movements and 60 frames of data. Dynamic CT images were resampled to 0.625 mm^3^ isotropic voxels. Due to scanner spatial resolution and longitudinal coverage limitations, static CT scans (120 kVp, 150 mA, 0.375 × 0.375 × 0.625 mm voxel size, and 0.531 pitch factor) were also performed to ensure the TMC joint was fully captured. Additionally, high-resolution peripheral quantitative CT images (HR-pQCT, XtremeCT II, Scanco Medical, Brüttisellen, Switzerland) were acquired to provide greater detail in the bony anatomy when selecting ALs. HR-pQCT scans were obtained using a standard in vivo imaging protocol (68 kVp, 1470 µA, 43 ms integration time, 900 projections/180°, 61 µm^3^ voxels) with an extended longitudinal coverage to capture the full TMC joint.

### Image processing

**Fig. 2:**
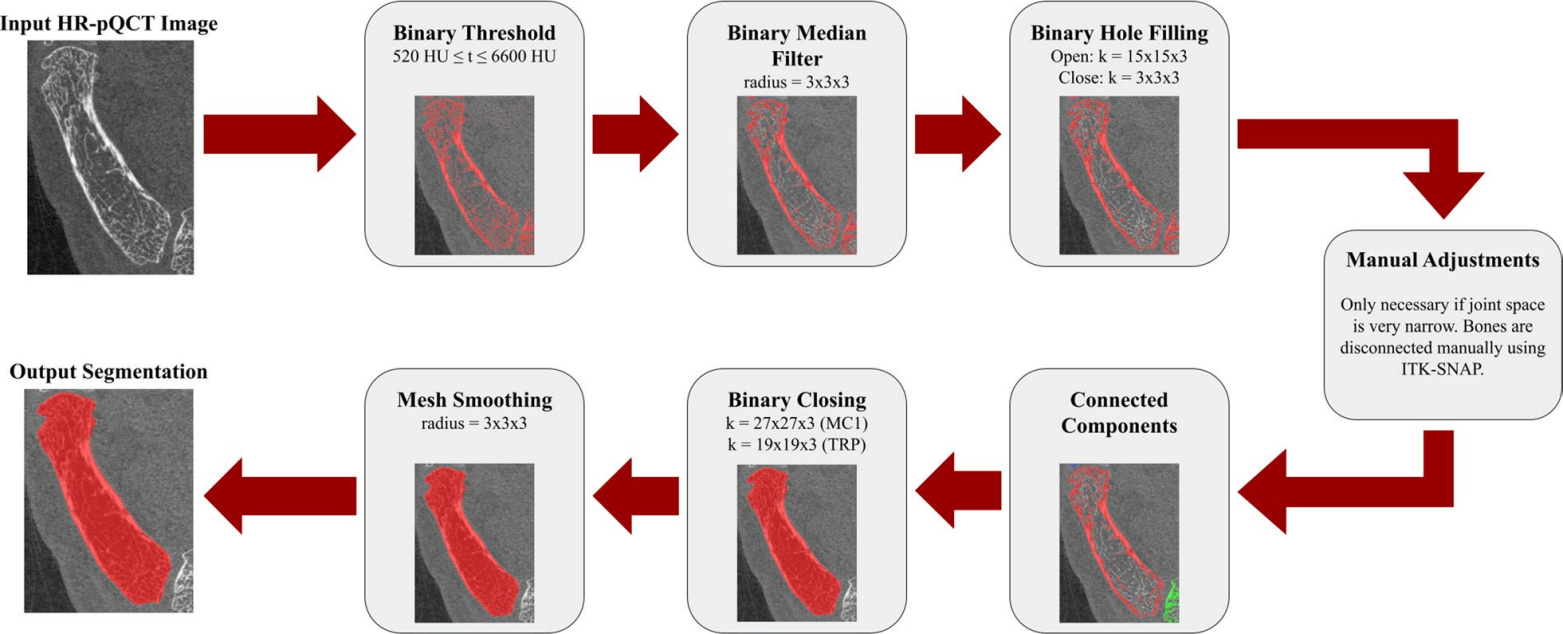
Processing pipeline for binarizing the TMC joint from HR-pQCT scans. The pipeline outlined is run for the first metacarpal and trapezium separately. The segmentation pipeline is semi-automated, only requiring manual intervention if the automated segmentation cannot differentiate between the first metacarpal and trapezium bones due to narrow joint space. The binary threshold (t) is provided in Hounsfield Units (HU) and the kernel size (k) for binary morphological operations are provided for the first metacarpal (MC1) and trapezium (TRP)

**Fig. 3:**
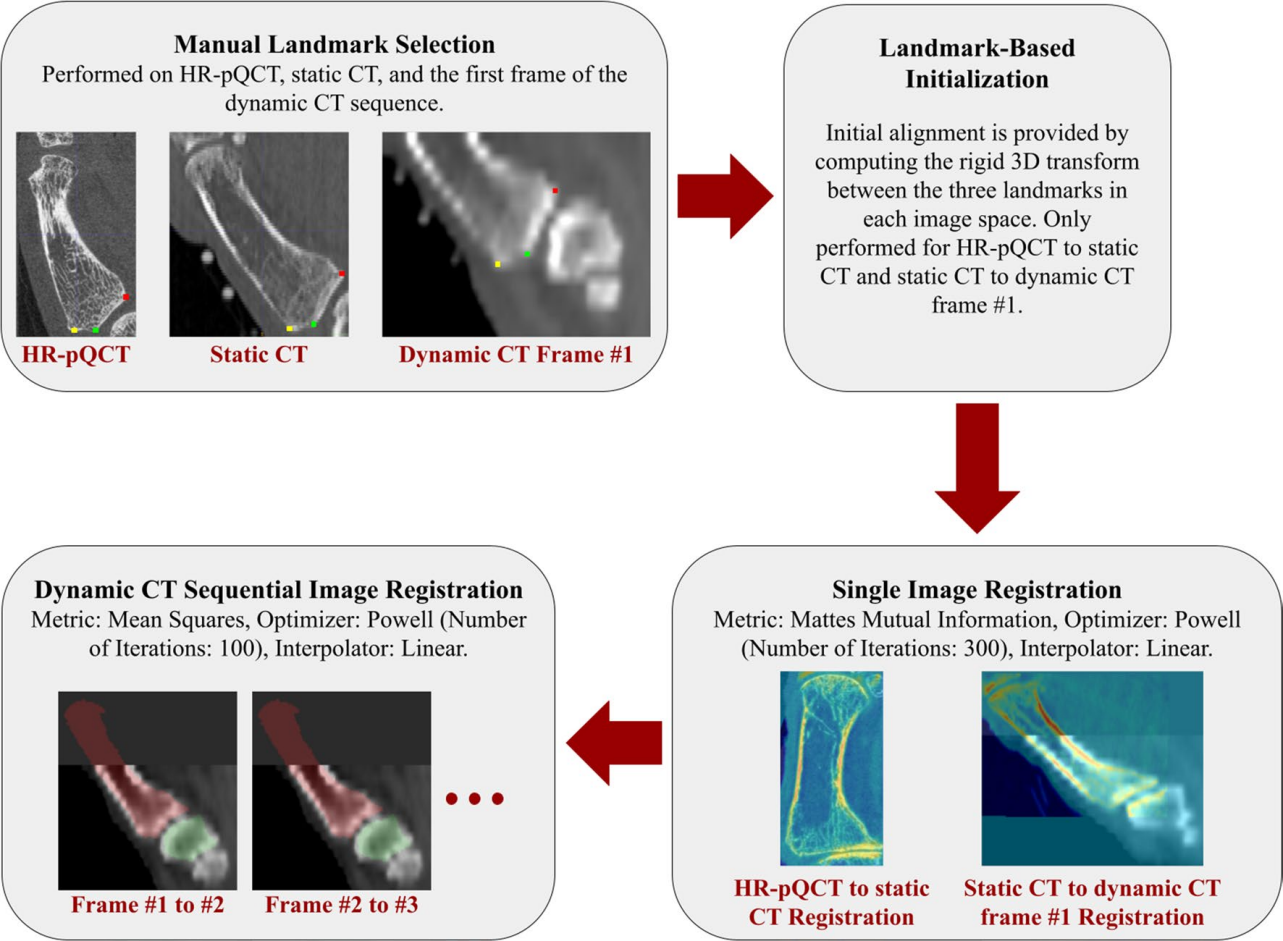
Overview of the image registration pipeline. Intensity-based registration is used to generate rigid, 3D transformation matrices that transform binary masks from the high-resolution CT scans to each frame of the dynamic CT dataset. Anatomical landmarks chosen in the high-resolution image space by each rater are also transformed to each dynamic CT frame using the same rigid transformation

All processing was performed using custom Python scripts (v.3.8.5) using the VTK (v8.2.0, Schroeder et al. [16]), ITK (v5.2.1, McCormick et al. [17]), and SimpleITK (v2.0.2, Lowekamp et al. [18]) libraries. All scripts used in this study are made available through a public GitHub repository (https://github.com/ManskeLab/DYNACT). Binary masks for the first metacarpal (MC1) and trapezium (TRP) were obtained from HR-pQCT images (Fig. 2). Then, a series of image registration steps were performed to move the bone masks to the dynamic CT space (Fig. 3). Each bone was processed individually and recombined in the dynamic CT image space.

#### Image segmentation

Grayscale HR-pQCT images were first binarized using a fixed global threshold (520 ≤ threshold ≤ 6600 Hounsfield units). Next, binary smoothing and a series of binary morphological operations (binary opening and closing) removed the binary components resulting from noise. A manual step was occasionally required to disconnect the MC1 and TRP bones when joint space was very narrow. Connected component labelling was then applied to isolate each bone. Further morphological operations and mesh smoothing were performed to obtain the final bone mask (Fig. 2). These bone masks were then used to select ALs.

#### Image registration

Bone masks and ALs were rigidly transformed to each frame of the dynamic CT scan in a three-step process (Fig. 3). This was required as the drastic resolution change from HR-pQCT to dynamic CT did not visually produce adequate alignment when registered directly. First, three manually placed landmarks were selected on each grayscale image (HR-pQCT, static CT, and the first frame of the dynamic CT scan) to generate an initial transformation between image spaces. Final image alignment was obtained automatically using intensity-based image registration between each set of images. Registration for HR-pQCT to static CT and static CT to the first dynamic CT frame used a Mattes Mutual Information metric [19], Powell optimizer, linear interpolator, and rigid 3D transform. Image registration between dynamic CT frames was performed using a sequential registration approach that utilized the position of the previous frame to provide initial alignment. A mean squares metric, Powell optimizer, linear interpolator, and rigid 3D transform were used to obtain binary masks and SCSs in each dynamic CT frame. Each registration provided a transformation matrix that could be used to transform the bone masks and ALs to the dynamic CT image space.

### Joint kinematics

#### Segment coordinate system definition

**Fig. 4:**
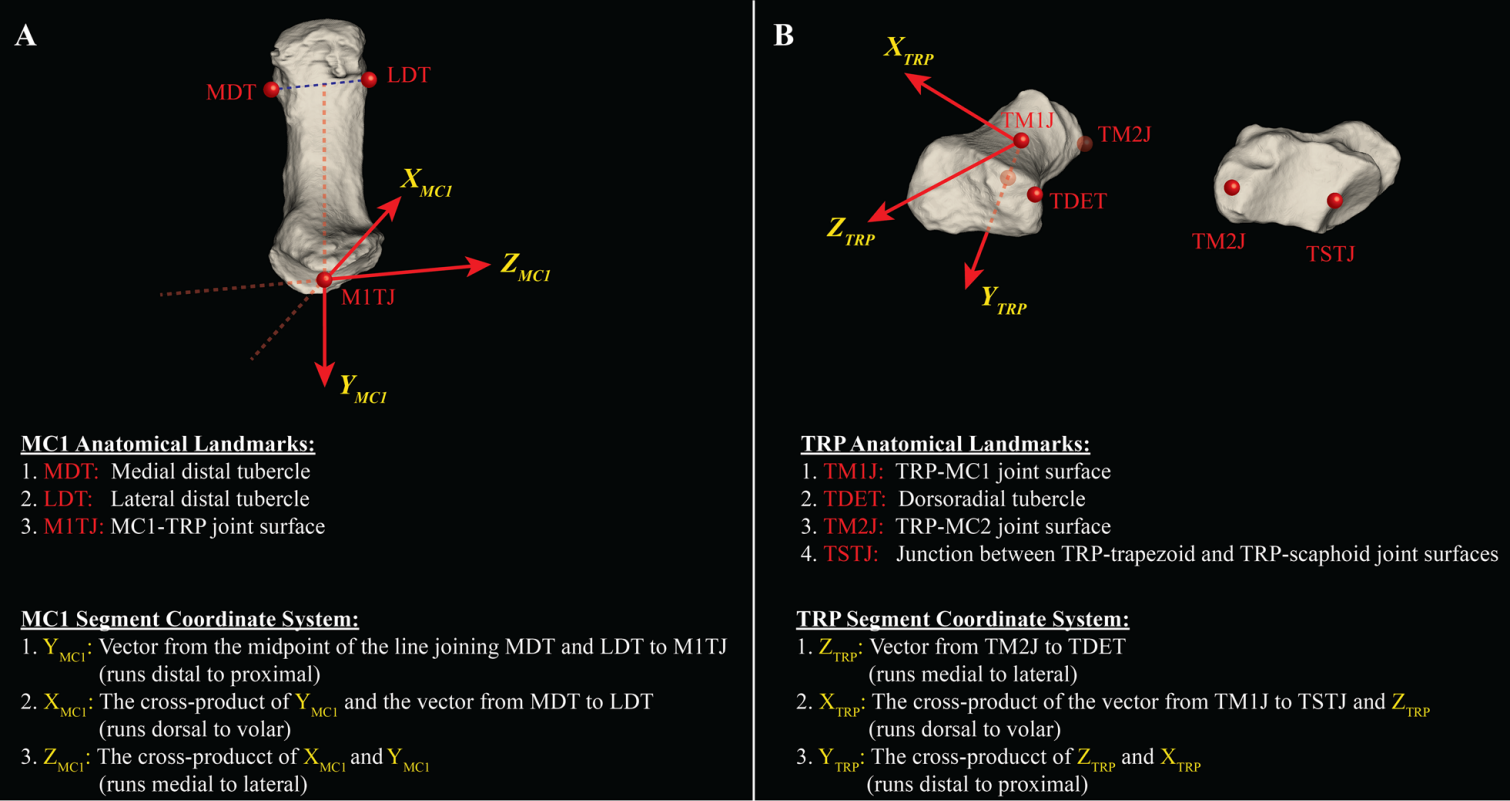
Visual representation of the anatomical landmarks (AL) used to define segment coordinate systems (SCS) on the first metacarpal (MC1) and trapezium (TRP) bones. **A** The three ALs required for the MC1 SCS, and **B** the four ALs required for the TRP SCS. Definitions of the SCS axes for each bone are provided below each figure. Similar figures were provided in the AL instruction document provided to each rater

Joint angles in the dynamic CT image space were computed using a joint coordinate system (JCS) representation [20] previously defined for the TMC joint that minimizes kinematic cross-talk [21] (Fig. 4). To establish a SCS, a custom visualization tool was developed using Python and VTK to visualize each bone model in 3D, allowing for AL selection. Three ALs were defined on the MC1: (1) LDT: centre of the lateral distal MC1 tubercle, (2) MDT: centre of the medial distal MC1 tubercle, and (3) M1TJ: centre of the MC1-TRP joint surface. The origin of the MC1 SCS was defined as M1TJ. Four ALs were defined on the TRP: (1) TM1J: centre of TRP-MC1 joint surface, (2) TM2J: centre of the TRP-second metacarpal joint surface, (3) TSTJ: centre of the junction between the TRP-trapezoid and TRP-scaphoid joint surfaces, and (4) TDET: distal end of the dorsoradial tubercle (viewed laterally). The origin of the TRP SCS was defined as TM1J. The definition of the SCSs for each bone have been previously described [21] and are shown in Fig. 4. ALs were later transformed to the dynamic CT image space where JCSs were defined in each frame.

#### Joint coordinate system definition

In accordance with a previous JCS definition in the TMC joint [21], the following mobile axis sequence was used: flexion–extension about the Z-axis of the TRP SCS (Fig. 4, Z_TRP_), abduction–adduction about the X-axis of the MC1 SCS (Fig. 4, X_MC1_), and axial rotation about a floating axis mutually orthogonal to Z_TRP_ and X_MC1_. ALs were transformed to each dynamic CT frame using transformation matrices obtained from the image registration process defined above (Fig. 3). Orientation of the MC1 relative to the TRP (${R}_{TRP\_MC1}$) was then computed by decomposing the ZYX mobile axis sequence:

$$R_{TRP\_MC1} = R_{{X_{MC1} }} \cdot R_{Y} \cdot R_{{Z_{TRP} }} = \left[ {\begin{array}{*{20}c} {cos\left( \gamma \right)cos\left( \beta \right)} & {sin\left( \gamma \right)cos\left( \alpha \right) + cos\left( \gamma \right)sin\left( \beta \right)sin\left( \alpha \right)} & {sin\left( \gamma \right)sin\left( \alpha \right) - cos\left( \alpha \right)cos\left( \gamma \right)sin\left( \beta \right)} \\ { - sin\left( \gamma \right)cos\left( \beta \right)} & {cos\left( \gamma \right)cos\left( \alpha \right) - sin\left( \gamma \right)sin\left( \beta \right)sin\left( \alpha \right)} & {sin\left( \alpha \right)cos\left( \gamma \right) + sin\left( \gamma \right)sin\left( \beta \right)cos\left( \alpha \right)} \\ {sin\left( \beta \right)} & { - sin\left( \alpha \right)cos\left( \beta \right)} & {cos\left( \beta \right)cos\left( \alpha \right)} \\ \end{array} } \right]$$

where $\alpha$ is the abduction–adduction angle (abduction is positive), $\beta$ is the axial rotation angle (internal rotation is positive), and $\gamma$ is the flexion–extension angle (flexion is positive). Joint translation was computed by determining the translation of the MC1 SCS origin (landmark M1TJ) with respect to the TRP SCS [22].

### Reproducibility and repeatability

Three raters (MTK, JJT, and TB) selected ALs on HR-pQCT bone masks to evaluate the reproducibility of the processing pipeline. One rater (MTK) selected ALs in three repeated trials on all images to evaluate repeatability. Each rater was provided with a document outlining the locations of each AL. The document described each AL, showing the surface on which to place the AL and examples of AL placement on one specimen. Each rater’s ALs were transformed to the dynamic CT image space to compute joint angles and translations and compare results between raters.

### Image registration accuracy

#### Experimental setup

As the post-processing pipeline used to quantify joint angles relied on intensity-based image registration, we estimated the accuracy of the image registration by computing target registration errors (TRE). Three fiducial markers (borosilicate beads, 4.76 mm diameter) were implanted into the MC1 bone of one specimen. An incision along the anatomical snuffbox was made and fiducial markers were implanted by drilling holes into the bone and gluing beads into place.

#### Target registration error

The 3D rigid transformation between image spaces defined by aligning the centroids of the fiducial markers was considered as the gold-standard transformation ($T_{GS}$). Fiducial markers were binarized manually using ITK-SNAP (v3.8.0) [23] and centroids of each fiducial marker in each image space were computed. Then, the TRE is defined as the difference between $T_{GS}$ and the transformation resulting from the intensity-based registration ($T_{IR}$), in a least squares sense. This calculation was performed over all $n$ voxels ($v_{i}$) of the moving image (Eq. 1).

1 $$TRE^{2} = \mathop \sum \limits_{i = 0}^{n} \left| {T_{GS} \left( {v_{i} } \right) - T_{IR} \left( {v_{i} } \right)} \right|^{2}$$

The accuracy of the TRE is limited by the accuracy of the gold-standard transformation. To estimate the error associated with the gold-standard transformation, the fiducial registration error ($FRE$) was first calculated using three manually selected landmarks on the bony geometry, referred to as targets. The root mean square (RMS) difference between the centroids of these targets and the fiducial markers provides an estimate of the *FRE* (Eq. 2), which is used to estimate the fiducial localization error ($FLE$) (Eq. 3) [24]. Finally, an estimate of the TRE for the $T_{GS}$ is calculated using the principal axes (PA) of the fiducial marker distribution (Eq. 4).

2 $$FRE^{2} = \sqrt {\frac{1}{{n_{f} }}\mathop \sum \limits_{i = 0}^{{n_{f} }} \left( {c_{i} - \widehat{{c_{i} }}} \right)^{2} }$$

3 $$FLE = \sqrt {\frac{{n_{f} }}{{n_{f} - 2}}} FRE$$

4 $$TRE^{2} \left( p \right) \approx \frac{{FLE^{2} }}{{n_{f} }}\left( {1 + \frac{1}{3}\mathop \sum \limits_{i = 1}^{3} \frac{{d_{i}^{2} }}{{f_{i}^{2} }}} \right)$$

where $n_{f}$ is the number of fiducial markers, $c_{i}$ is the centroid of each fiducial marker, $\widehat{{c_{i} }}$ is the centroid of the target, $p$ is the target location, $d_{i}$ is the distance from the centroid of the target to the PA, and $f_{i}$ is the RMS distance from each fiducial marker’s centroid to the PA.

### Statistical analysis

All statistical analyses were performed using R (v.4.1.1) [25] and SPSS (v.28.0.0.0) [26]. Inter- and intra-rater reliability coefficients (ICC), Bland–Altman analysis, and frame-by-frame RMS error (RMSE) of joint angles between raters were calculated to evaluate reproducibility and repeatability. Inter-rater ICC was modelled as a two-way, random effects, absolute agreement, single measurement model. Intra-rater ICC was modelled as a two-way, mixed effects, absolute agreement, single measurement model. Agreement between and within raters was interpreted as follows: excellent agreement (ICC ≥ 0.9), good agreement (0.75 ≥ ICC > 0.9), moderate agreement (0.5 ≥ ICC > 0.75), and poor agreement (ICC < 0.5) [27]. Bland–Altman analysis was performed by comparing each rater’s angle results to the mean angles between all raters. A linear regression of each rater’s Bland–Altman results was used to evaluate the slope of each rater’s angles compared to the mean. As a secondary analysis, frame-by-frame RMSE of joint translations were calculated for each spatial plane.

To evaluate the error in SCS definition between raters, the orientation difference between SCS axes was calculated between each pair of raters. First, the dot product between corresponding SCS axes was computed (*e.g.*, $X_{SCS\_1} \cdot X_{SCS\_2}$, $X_{SCS\_1} \cdot X_{SCS\_3}$, etc.) in the HR-pQCT image space and again in each of the 60 dynamic CT frames. Then, for each SCS axis and for each pair of raters, the RMSE between the HR-pQCT SCS orientation difference and each dynamic CT SCS orientation difference was computed, resulting in 60 RMSE values. This process was then repeated for each specimen.

## Results

### Joint kinematics

To compare the TMC joint range of motion with previous studies, the mean joint angle across all specimens and across all raters is provided along with the angle range. Mean joint angles were 40.7° ± 5.8° (range: 20.0° to 53.2°) for abduction–adduction, −16.3° ± 7.1° (range: −28.4° to −5.2°) for flexion–extension, and -5.4° ± 4.1° (range: -13.2° to 4.6°) for axial rotation.

### Joint kinematics reproducibility and repeatability

**Fig. 5:**
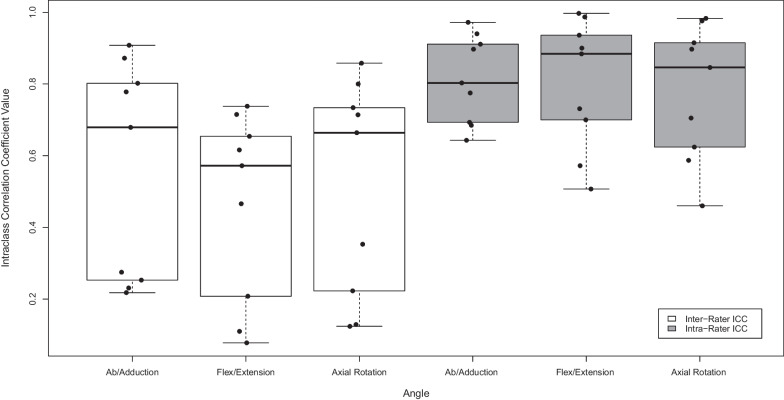
Box plot of inter- and intra-rater intraclass correlation coefficients (ICC) for each measured angle, across all specimens (n = 9). Inter-rater ICCs were calculated using a two-way, random effects, absolute agreement model. Intra-rater ICCs were calculated using a two-way, mixed effects, absolute agreement model. Intra-rater ICC values showed better agreement than inter-rater ICC values

**Fig. 6:**
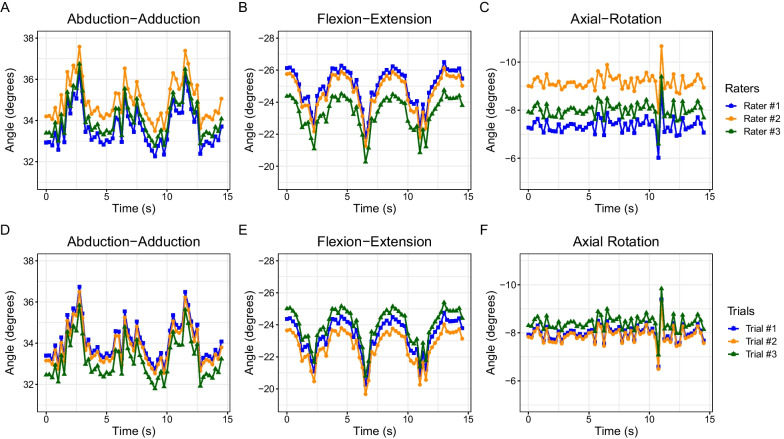
Joint angle results for one specimen. Dynamic CT scans were acquired with a gantry rotation of 4 volumes/second and a scan time of 15 s, resulting in 60 data points per movement. Inter-rater plots are shown in the top row and intra-rater plots are shown in the bottom row. **A** Inter-rater abduction-adduction angles; **B** inter-rater flexion-extension angles; **C** inter-rater axial-rotation angles; **D** intra-rater abduction-adduction angles; **E **intra-rater flexion-extension angles; and **F** intra-rater axial-rotation angles

**Fig. 7:**
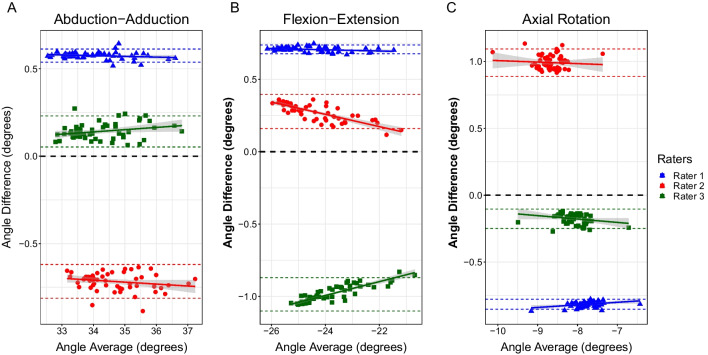
Sample Bland–Altman plots for one specimen comparing joint angles from each rater to the average angle between all three raters. **A** Abduction–adduction angles; **B** flexion–extension angles; and **C** axial-rotation angles

**Fig. 8:**
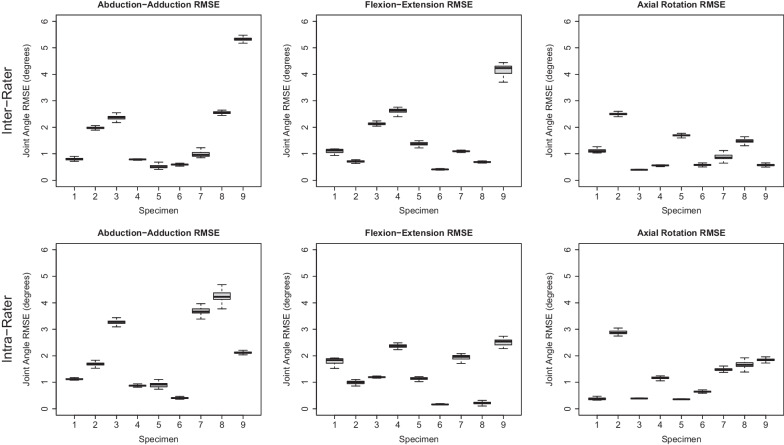
Box plots showing the inter- and intra-rater root mean square error (RMSE) of joint angles across all specimens (n = 9). RMSE values were computed across each dynamic CT frame, resulting in 60 RMSE values for each specimen

Intra-rater ICC values had the following ranges across all specimens: abduction–adduction: 0.64–0.91, flexion–extension: 0.50–0.99, axial rotation: 0.46–0.98. Inter-rater ICC values had the following ranges: abduction–adduction: 0.21–0.90, flexion–extension: 0.07–0.71, axial rotation: 0.12–0.85. All ICC values are presented in Fig. 5. A comparison of joint angles from one specimen are shown in Fig. 6. Results of the Bland–Altman analysis for one specimen are shown in Fig. 7. While roughly half of the slopes were statistically significantly different than zero, all regression lines had slopes with small values (< 0.06, absolute value). Average RMSEs are presented in Fig. 8 and intra-rater RMSE tended to be lower than inter-rater RMSE. Average inter-rater RMSE across specimen was 1.76° ± 1.54° for abduction–adduction, 1.58° ± 1.19° for flexion–extension, and 1.08° ± 0.69° for axial rotation. Average intra-rater RMSE across specimen was 2.03° ± 1.38° for abduction–adduction, 1.36° ± 0.84° for flexion–extension, and 1.19° ± 0.85° for axial rotation. The following joint translation RMSEs were computed: X < 1.44 mm, Y < 1.55 mm, Z < 2.15 mm for inter-rater RMSE, and X < 1.26 mm, Y < 0.83 mm, Z < 1.05 mm for intra-rater RMSE.

### Image co-registration accuracy

TREs for the manually selected targets were found to be 0.0010, 0.0010, and 0.0011 mm for the registration between HR-pQCT and static CT, 0.64, 0.43, and 0.66 mm for the registration between static CT and the first dynamic CT frame, and 0.33, 0.33, and 0.27 mm for the registration between the first and second dynamic CT frame. The error associated with the gold-standard transformation (*i.e.*, using the centroids of the implanted fiducial markers) was found to be 0.00049 mm for the image registration between HR-pQCT and static CT, 0.31 mm for image registration between static CT and the first frame of the dynamic CT dataset, and 0.62 mm between the first and second dynamic CT frames.

When analyzing the orientation difference between each bone’s SCS axes in the HR-pQCT and dynamic CT image space, we found that the RMSE between image spaces for each dynamic CT frame, for all pairs of raters, was less than $1.3{e}^{-11}$ mm (data not shown).

## Discussion

We propose a semi-automated pipeline to quantify joint motion in the TMC joint using dynamic CT. Our joint angle results (Table 1) fall within range of previously reported TMC joint angles for similar thumb movements [1, 21, 28]. While much of this pipeline has been automated, SCSs were defined using manually placed ALs. Contrary to our original hypothesis, we found good repeatability (*i.e.*, intra-rater) and moderate reproducibility (*i.e.*, inter-rater) for joint angle computation. Additionally, we found joint translational errors to be lower when ALs were selected by a single rater as compared to ALs selected by multiple raters, for each plane of motion. However, we found that within rater results exhibited lower variability (Fig. 5, Intra-Rater ICC) compared to between rater results (Fig. 5, Inter-Rater ICC) for all joint angles. Inter-rater flexion–extension angles demonstrated lower ICC values when compared to inter-rater abduction–adduction angles. Trapezium morphology has been shown to change with age and disease state [29], which may lead to greater variability between AL placement and explain the decreased inter-rater ICC for the flexion–extension angles. In previous studies, automated methods have been developed to define joint SCSs based on joint surface geometry in the TMC joint [30], and use statistical shape modelling (SSM) to estimate lower limb geometry and pose [31]. While such methods may improve the reproducibility of SCS definition in healthy TMC joints, it is not yet clear how robust such automated methods are to changes in articular morphology, particularly in diseased TMC joints. In a *post-hoc* qualitative analysis, we found that joints with considerable joint degeneration had poorer agreement between raters compared to joints that appeared healthy. In joints that visually appeared healthy, ICC values were moderate to excellent between and within raters (ICC > 0.5). These results indicate that manual selection of ALs to compute TMC joint kinematics may be appropriate for healthy joints. While our results indicate only moderate reproducibility in joint angle computation using manually placed ALs, the use of automated approaches for SCS definition [30] for diseased TMC joints warrants further investigation.

**Table 1:** Mean joint angles across three raters

Specimen	Joint angle mean [Range] (degrees)		
	Abduction–adduction	Flexion–extension	Axial rotation
1	34.2 [32.8, 36.9]	−24.3 [−25.8, −21.1]	−8.2 [−9.6, −6.8]
2	43.4 [41.1, 45.7]	−17.5 [−19.8, −16.0]	−4.4 [−6.5, −2.6]
3	46.9 [44.3, 48.6]	−11.9 [−12.8, −10.3]	−10.6 [−12.9, −9.7]
4	46.3 [43.7, 49.8]	−3.1 [−5.2, −1.0]	−10.4 [−13.2, −7.9]
5	33.8 [30.7, 37.8]	−13.8 [−15.1, −9.5]	−1.9 [−3.3, 0.7]
6	38.2 [36.5, 41.6]	−20.3 [−21.6, −18.2]	1.7 [−0.8, 4.6]
7	40.4 [36.9, 47.8]	−18.1 [−24.1, −15.1]	−7.5 [−9.6, −4.4]
8	48.7 [46.6, 53.2]	−11.5 [−13.5, −10.0]	−1.9 [−4.0, 0.5]
9	34.4 [20.0, 40.1]	−26.2 [−28.4, −20.1]	−5.9 [−7.9, −1.5]

In Bland–Altman analysis, we compared each rater’s resulting joint angles to the mean across all raters. Applying a linear regression model to each rater’s Bland–Altman plot revealed very small slopes across all raters and specimens (Table 2). While approximately half of these slopes were statistically significantly different than zero (*p* < 0.05), the small magnitude of regression slopes indicates that the difference between each rater and the average was not related to the magnitude of the average angle measurement. Further, we found that there was consistent bias between raters and the mean (Fig. 7), representative of expected differences between rater’s AL placement. Whether differences detected between raters is clinically relevant warrants further investigation.

**Table 2:** Slopes of regression lines plotted for Bland–Altman analysis

Specimen	Abduction–adduction Slope			Flexion–extension slope			Axial rotation slope		
	Rater 1	Rater 2	Rater 3	Rater 1	Rater 2	Rater 3	Rater 1	Rater 2	Rater 3
1	**0.03**	−**0.01**	−**0.02**	−**0.02**	**0.02**	**0.01**	−**0.01**	−0.02	0.01
2	**0.01**	**0.01**	−0.02	−**0.01**	**0.02**	**0.01**	0.00	−0.04	0.00
3	−**0.03**	−**0.02**	−0.02	**0.04**	**0.03**	**0.00**	−**0.02**	−0.01	−0.01
4	0.00	0.05	0.01	**0.00**	−**0.05**	0.04	−**0.01**	−**0.01**	−**0.05**
5	0.00	−0.01	0.01	0.00	**0.02**	−**0.04**	−0.01	−**0.01**	−0.01
6	**0.02**	−**0.01**	**0.02**	−0.01	0.00	−0.01	−**0.01**	**0.01**	0.00
7	−0.01	**0.00**	−0.02	0.01	−**0.01**	−0.01	0.00	0.01	0.00
8	**0.02**	−**0.01**	−**0.02**	−0.04	0.00	0.01	**0.01**	**0.00**	−**0.02**
9	**0.06**	−**0.01**	−**0.03**	0.00	−0.01	0.06	−**0.06**	**0.01**	−**0.04**

Bold indicates statistical significance, where *p* < 0.05

The image processing required for dynamic CT scans introduces new sources of error that are not prevalent in motion capture systems. In this study, intensity-based image registration was first performed between the HR-pQCT and dynamic CT image spaces. However, we were not able to determine an optimal set of registration parameters that visually produced adequate bone alignment. This was likely due to the drastic change in spatial resolution between the HR-pQCT and dynamic CT image spaces. With the addition of an intermediate registration step using static CT scans of the specimens, we were able to obtain improved bone alignment when assessed visually. Further, quantitative analysis of the TREs associated with each image registration found voxel or sub-voxel level errors, suggesting that only a small portion of the error in the joint angle results between raters was due to the image registration steps. When comparing SCS orientation between raters in the HR-pQCT and dynamic CT image space, we found little difference in the orientation of SCS axes between image spaces. This suggests that any orientation difference between SCSs defined in the HR-pQCT image space are held constant after transforming all data to the dynamic CT space, further reinforcing our confidence that the resulting difference in joint angles and translations between raters are due to rater biases when defining SCSs, not due to the image registration.

This study had several limitations. First, the dynamic CT image quality prevented the direct image segmentation and SCS definition. Our scanner was limited to a spatial resolution of 0.625 × 0.625 × 2.5 mm and a longitudinal coverage of 40 mm, not enough to fully capture the MC1 bone; however, newer scanners have dynamic protocols with larger longitudinal coverage and improved spatial resolution (*e.g.*, 160 mm longitudinal coverage, 0.625 mm^3^ spatial resolution). We utilized HR-pQCT scans to obtain full joint images for initial image segmentation. However, this step poses challenges in vivo due to the long scanning time and increased exposure to ionizing radiation. Instead, a high-resolution static CT scan that includes the whole bone can be obtained from other clinical CT scanners that are widely available. For example, recent developments in cone beam CT scanners allows for imaging of the hand at resolutions up to 0.2 mm^3^ (*e.g.*, CurveBeam HiRise weight-bearing CT scanners), which may be sufficient for providing a more detailed initial segmentation than images obtained from dynamic CT. Despite the additional image processing steps used here to overcome the scanner limitations, the TRE results indicate there was no substantial impact on joint angles. Second, the TMC joints in the acquired specimens varied in joint condition. Some specimens had considerable degeneration *(e.g*., joint space narrowing, presence of osteophytes, etc.) and others appeared healthy. We found that joint angle RMSE results were as high as 5.33° and 4.25° for inter-rater and intra-rater abduction–adduction, respectively (Fig. 8). However, the degree to which error is influenced by differences between healthy and diseased joints requires further study. Third, while we found that repeatability was higher than reproducibility (Fig. 5, 6), it is worth noting that the repeatability analysis was only performed using one rater. This rater was considered the most experienced in terms of TMC joint anatomy among the raters in this study and therefore our improved repeatability results may reflect this. Finally, the workflow we present in this study is semi-automated, with manual corrections only needed during image segmentation when joint space is narrow. However, employing more advanced methods for image segmentation (*i.e.*, statistical shape modelling approaches [32]) may be incorporated to completely automate the post-processing of dynamic CT datasets and further reduce processing time.

## Conclusions

This work has shown that our image processing pipeline to quantify joint angles and translations from dynamic CT scans of the TMC joint is fast, repeatable, but only moderately reproducible. We found poor reproducibility of joint angle computation using our pipeline in joints that appeared diseased or had severe joint degeneration. If ALs are to be placed manually on dynamic CT datasets of diseased joints, we recommend placement of ALs by a single rater. Moreover, we recommend the use of a detailed document to ensure consistency when placing ALs. Future work will be performed to determine whether the pipeline is sufficiently sensitive to differentiate between healthy and diseased TMC joint kinematics. Moreover, the clinical significance of the difference in joint angles and translations found in this work between raters warrants further investigation. Many dynamic CT studies process their datasets using closed-source software that is not widely available (*e.g.*, Materialise Mimics). Our open-source pipeline to quantify joint angles from dynamic CT scans requires approximately 1.5 h to completely process a single movement. Finally, this pipeline can be easily implemented for different motions, joints, and scan acquisition parameters, and can therefore enhance the utilization of dynamic CT to assess joint kinematics.

## Data Availability

All scripts, code, and software used in this publication are available on GitHub: https://github.com/ManskeLab/DYNACT. The datasets used and/or analyzed during the current study are available from the corresponding author on reasonable request.

## Abbreviations

3D: Three dimensional
AL: Anatomical landmark
CT: Computed tomography
FLE: Fiducial localization error
FRE: Fiducial registration error
HR-pQCT: High resolution peripheral computed tomography
ICC: Intraclass correlation coefficient
JCS: Joint coordinate system
LDT: Lateral distal tubercle
M1TJ: First metacarpal trapezium joint
MC1: First metacarpal
MDT: Medial distal tubercle
RMS: Root mean square
RMSE: Root mean square error
SCS: Segment coordinate system
TM1J: Trapezium first metacarpal joint
TM2J: Trapezium second metacarpal joint
TMC: Trapeziometacarpal
TRE: Target registration error
TRP: Trapezium
TSTJ: Trapezium, scaphoid, trapezoid junction
